# The effect of Chinese Jinzhida recipe on the hippocampus in a rat model of diabetes-associated cognitive decline

**DOI:** 10.1186/1472-6882-13-161

**Published:** 2013-07-06

**Authors:** Xiao-Hui Chang, Li-Na Liang, Li-Bin Zhan, Xiao-Guang Lu, Xiang Shi, Xin Qi, Zhao-Lan Feng, Mei-Juan Wu, Hua Sui, Lu-Ping Zheng, Fu-Liang Zhang, Jie Sun, Chang-Chuan Bai, Nan Li, Guo-Zhu Han

**Affiliations:** 1 Department of Traditional Chinese Medicine, the Second Affiliated Hospital, Dalian Medical University, Liaoning, China; 2 Department of Traditional Chinese Medicine and Western Medicine, Institute of Integrated Medicine, Dalian Medical University, Liaoning, China; 3 Department of Emergency Medicine, Zhongshan Hospital, Dalian University, Dalian, Liaoning, China; 4 Department of Physiology and Biophysics, Case Western Reserve University School of Medical Science, Cleveland, OH, USA; 5 Institute of Public Hygiene, Dalian Medical University, Liaoning, China; 6 Outpatient Department of Dalian Traditional Chinese Medicine Institute, Liaoning, China; 7 Institute of Chemical Technology, Dalian University of Technology, Liaoning, China; 8 Institute of Medicine, Dalian Medical University, Liaoning, China

**Keywords:** Diabetes, Cognitive decline, Step down test, Morris water, Immunobloting analysis, Hippocampus

## Abstract

**Background:**

To investigate the effects of treatment with Multi component Chinese Medicine Jinzhida (JZD) on behavioral deficits in diabetes-associated cognitive decline (DACD) rats and verify our hypothesis that JZD treatment improves cognitive function by suppressing the endoplasmic reticulum stress (ERS) and improving insulin signaling transduction in the rats’ hippocampus.

**Methods:**

A rat model of type 2 diabetes mellitus (T2DM) was established using high fat diet and streptozotocin (30 mg/kg, ip). Insulin sensitivity was evaluated by the oral glucose tolerance test and the insulin tolerance test. After 7 weeks, the T2DM rats were treated with JZD. The step-down test and Morris water maze were used to evaluate behavior in T2DM rats after 5 weeks of treatment with JZD. Levels of phosphorylated proteins involved in the ERS and in insulin signaling transduction pathways were assessed by Western blot for T2DM rats’ hippocampus.

**Results:**

Compared to healthy control rats, T2DM rats initially showed insulin resistance and had declines in acquisition and retrieval processes in the step-down test and in spatial memory in the Morris water maze after 12 weeks. Performance on both the step-down test and Morris water maze tasks improved after JZD treatment. In T2DM rats, the ERS was activated, and then inhibited the insulin signal transduction pathways through the Jun NH2-terminal kinases (JNK) mediated. JZD treatment suppressed the ERS, increased insulin signal transduction, and improved insulin resistance in the rats’ hippocampus.

**Conclusions:**

Treatment with JZD improved cognitive function in the T2DM rat model. The possible mechanism for DACD was related with ERS inducing the insulin signal transduction dysfunction in T2DM rats’ hippocampus. The JZD could reduce ERS and improve insulin signal transduction and insulin resistance in T2DM rats’ hippocampus and as a result improved the cognitive function.

## Background

The prevalence of type 2 diabetes mellitus (T2DM) is increasing rapidly in many industrialized nations, especially in China [1]. Recently, there has been growing apprehension about the complications of diabetes, especially diabetes-associated cognitive decline (DACD). DACD, also called diabetic encephalopathy (DE) [2], represents a complication of the diabetic brain and manifests as a gradual decline of cognitive function. Although the exact mechanisms of DACD have not been fully elucidated, it is known that impaired insulin signaling transduction involved in the metabolism of Amyloid Protein (Aβ) and tau [3] plays an important role in DACD. The activity of Jun NH2-terminal kinase (JNK) protein is significantly elevated in various tissues in T2DM patients and animal models, which can impair insulin signaling transduction and lead to insulin resistance through activating the serine phosphorylation of insulin receptor substrate-1 (IRS-1) [4]. Meanwhile, JNK is an important downstream signal of the endoplasmic reticulum stress (ERS) response. Under stressful conditions, three transmembrane endoplasmic reticulum signaling proteins then dissociate from molecular chaperone 78-kD glucose-regulated protein (GRP78/Bip) and activate JNK [5]. Recently, several reports about Alzheimer’s Disease (AD) support a mechanistic connection between ERS and AD [6-8], which had similar pathology changes, but whether ERS plays a role in DACD is not clear. We hypothesized that ERS-induced JNK activation impairs insulin signaling transduction, resulting in DACD in the hippocampus of T2DM rats.

Currently, there are no effective treatments that can prevent the development of DACD in T2DM. Traditional Chinese Medicines (TCMs) have been used for centuries in China in the prevention and treatment of many diseases and have proven to be effective and safe for clinical use. As a result, they have attracted global attention [9,10]. May and his colleagues [11] published a systematic review of the effectiveness and safety of TCMs for treating early dementia. They concluded that Chinese herbs could improve cognitive and memory impairment and could be useful in the treatment and prevention of early dementia, including AD.

Multi-component Chinese medicine theory is a new Traditional Chinese Medicines theory system, which was first proposed by the Tianjin University of Traditional Chinese Medicine Zhang Boli professor [12]. It followed the principle of Chinese medicine prescription compatibility principle, application of effective components or effective parts extracts compatibility formula. The Multi component Chinese Medicine Jinzhida (JZD) from green tea, ginseng and polygala are complex extracts made of theanine, tea polyphenols, ginsenosides and polygalic acid and were designed according to the theory of Multi-component Chinese medicine. Each of these substances therefore has been shown to have neuroprotective functions, and each has a clear mechanism of action [12-14]. These compounds are compatible when taken together, and it is thought that their concurrent use may amplify the therapeutic efficacies of each component. This results in maximal therapeutic efficacy with minimal adverse effects, similar to the results of “cocktail therapies” for AIDS [15]. We therefore sought to evaluate the effects of these compounds in combination on DACD by treating streptozotocin-induced DACD rats with JZD.

## Methods

### Preparation of multi component Chinese medicine Jinzhida

Multi component Chinese Medicine Jinzhida (JZD) are complex extracts derived from tea polyphenols, theanine,ginsenosides and polygalic acid, which were mixed in a blender in a ratio of 3:3:2:1 by weight. Tea polyphenols were purchased from Wufeng Tianjian Plant Products Ltd. (Hubei Province, China), theanine from Jintan City Qianyao Pharm Co. Ltd. (Jiangsu Province, China), ginsenosides from Fusong County Natural Biotechnology Ltd. (Jilin Province, China) and polygalic acid from Jingyue Plant and chemical technology Ltd. (Sanxi Province, China) They were dissolved in double-distilled water and concentrated to 39 mg/kg, 104 mg/kg and 390 mg/kg for the low, middle and high dose groups, respectively.

### Animals

All animal experiments were conducted in accordance with the NIH Principles of Laboratory Animal Care and the institutional guidelines for the care and use of laboratory animals at Dalian Medical University(Dalian, China). Eight weeks-old male Sprague–Dawley (SD) rats weighing 200 ± 20 g were obtained from the Experimental Animal Center at Dalian Medical University, Dalian City, Liaoning Province, China, license number: SCXK (Liaoning Province) 2008–0002. Rats were allowed to acclimatize in an environmentally-controlled room (with a temperature of 22 ± 3°C and humidity of 55 ± 5%) with an alternating 12 h light/dark cycle and free access to chow and water. After 3 days acclimation, rats in the T2DM group were fed a high-fat diet (40% fat, 25% protein and 35% carbohydrate, as a percentage of total kcal; Anlimo Technology, Nanjing, China) [16] and in the control group were fed a standard diet. After 4 weeks of a high-fat diet, rats were fasted for 12 h (with free access to water), and each rat was injected intraperitoneally with 30 mg/kg streptozotocin (STZ; Sigma, St. Louis, MO, USA) in 0.1 M citrate buffer solution, pH 4.4. Rats in the control group were injected with the citrate buffer solution [17]. Random blood sugar (RBS) levels were measured in blood obtained by clipping the tails of the rats. A measurement of ≥16.7 mmol/L 3 days after STZ injection was considered diagnostic of T2DM [18].

After streptozotocin injection, fasting serum insulin (FSI) levels were obtained, and glucose tolerance tests (OGTT) and insulin tolerance tests (ITT) were performed. Samples for FSI were taken from the angular vein and analyzed using a radioimmunoassay kit (Atom High-tech, Beijing, China). Glucose (50% [wt/wt]; 2 g/kg, given orally) and regular human insulin (0.75 U/kg; Novo Nordisk, Tianjin, China) were used in the OGTT[19] and ITT [20], respectively.

### Experimental groups and treatments

A total of 28 rats that developed T2DM and had comparable body weights were chosen and randomly divided into three treatment groups and a DACD model group as described in Table 1. Rats in the three treatment groups received different concentrations of JZD 1 ml/100 g orally twice a day for 5 weeks. The DACD model (Mod) and control (Cont) group rats received isotonic Na chloride as control injections.

**Table 1:** Experimental groups and treatments

**Group**	**N**	**Type of animals**	**Treatment**
Cont	10	Normal rats	Isotonic Na chloride
Mod	7	T2DM rats	Isotonic Na chloride
LJZD	7	T2DM rats	Low-dose JZD
MJZD	7	T2DM rats	Middle-dose JZD
HJZD	7	T2DM rats	High-dose JZD

### Behavioral experiments

Behavioral tests, including Step-down tests and Morris water maze tests, were conducted during the last 7 days of treatment according to the following schedule: Day 1–2 step-down test and Day 3–7 Morris water maze test. The rats were subsequently euthanized.

### Step-down test

The training apparatus was a 60 cm × 20 cm × 20 cm plastic box, the floor of which was made of parallel 0.1 cm-caliber stainless steel bars spaced 0.5 cm apart. An elevated rubber platform (diameter: 10 cm, height: 4.5 cm) was placed by the left wall of the training box apparatus. On the first training day, rats were first exposed to a 5-min learning course, during which they were permitted to move freely through the chamber, and were then placed on the platform. If the animals stepped down from the platform (“error trial”), they were punished by an electric foot shock (36 V, AC). The numbers of “errors” (steps down from the platform) during the training period were recorded. After 15 minutes of training, the rats were placed on the platform and the latency to step-down was recorded as the 1st learning grade (D1 latency). After 24 h, the latency was assessed again and recorded as the 2nd learning grade (D2 latency), which was taken as a measure of memory retention.

### Morris water maze

The apparatus consisted of a circular pool (50 cm height × 120 cm diameter) filled with 26 ± 1°C water made opaque with milk. A clear Plexiglas platform (29 cm high × 9 cm diameter) was submerged 1 cm below the water surface. The pool was divided into four equal quadrants and each quadrant was marked by a different visual cue. The platform was randomly placed in one quadrant for the duration of the experimental procedure. On the first day, rats were given a 120 s habituation session in the pool without the platform. On the following 4 days, each rat received four 120 s learning trials with the platform, with 60 s resting periods between trials. For each learning trial, rats were placed into the water facing the pool wall at one of four points of entry. The escape latency (time needed to locate the submerged platform) was recorded for each trial. If a rat was unable to locate the platform within 120 s, it was led to the platform and allowed to rest there for 60 s. The escape latency in these cases was recorded as 120 s. After completion of the learning trials, the platform was removed from the pool. Each rat was then subjected to a 120 s memory retention test, in which the amount of time and frequency the rat spent swimming in the same quadrant as the platform had previously been hidden was recorded [18].

### Sample preparation

After behavioral tests, the animals were anesthetized with intraperitoneal injection of 4% chloral hydrate and euthanized. Brain structures were removed and stored at −80°C until further use. The hippocampus was then isolated for protein preparation. Briefly, based on histological observations, the border of the selected region was identified in the cross section of the frozen specimen and was marked gently with an autoclaved surgical knife. According depending on the size of the region of interest, eight to sixteen sections (10 μm per section) were used for protein preparation. Tissues were collected with an autoclaved pipette tip and placed immediately into 25 μl of pre-chilled 2× Laemmli SDS sample buffer (0.125 M Tris–HCl pH 6.8, 4% SDS, 20% v/v glycerol and 0.2 M DTT) [21]. Samples were subjected to ultrasound for 10 min in pre-chilled water, then boiled and denatured for 5 min. The sample was then maintained at 4°C and centrifuged at 15,000 rpm for 5 min, after which the supernatant was collected. Protein quantification was performed using Coomassie protein assay reagent (Pierce Biotechnology, Rockford, IL, USA) and absorbance measured at 595 nm with a Bradford protein assay and using bovine serum albumin as the protein standard.

### Western blot analysis

All chemicals for western blot analysis purchased from Sigma-Aldrich.Shanghai Ltd. (Shanghai, China). For each sample, 50 μg of protein was loaded in each lane were separated in a 8-12% SDS-polyacrylamide gel and transferred to a nitrocellulose membrane (Bio-Rad, CA). The membrane was blocked with 5% skim milk in PBS for 3 h and washed in Tris-buffered saline three times. The membrane was then incubated overnight at 4°C with one of the following primary antibodies: anti- phosphorylated protein kinase RNA-like endoplasmic reticulum kinase (anti-PERK) 1:1,000, anti-Jun NH2-terminal kinase (anti-JNK) 1:1,000 (Santa Cruz, USA), anti- inositol-requiring enzyme-1(anti-IRE1) 1:1,000, anti-α subunit of translation initiation factor 2 (anti-eIF2α) 1:500, antiphospho-eIF2α 1:500, antiphospho-JNK 1:1,000, antiphospho-PERK 1:800 , anti-protein kinase B (anti-AKT) 1:1,000 or antiphospho-AKT 1:800 (Cell Signaling, USA), antiphospho-IRE1 1:800 (Abcam, UK), anti-78-kD glucose-regulated protein (anti-GRP78) 1:800 (Stressgen, CA), anti-insulin receptor substrate-1 (anti-IRS-1) 1:1,000 , antiphospho-IRS-1 (ser307) 1:1,000 (Upstate, USA) and monoclonal anti-β actin (Sigma, USA). After incubation with primary antibody, the membrane was then incubated for 3 h at room temperature with an anti-rabbit or an anti-mouse IgG HRP-linked antibody (GE Healthcare, UK) at a dilution of 1:2,500. Immunoreactive bands were detected using the Lumi-Light Western Blotting Substrate (Roche, USA). The intensity of bands was measured with an imaging system (UVP, USA) coupled with LabWorks 4.6 analysis software. The relative intensities were calculated by dividing by anti-β actin. The bands were analyzed according to the relative density of each antibody, which is obtained by dividing the density of each antibody value by the internal reference β-Actin.

### Statistical analysis

Data were expressed as means ± SD. Statistical significance was analyzed using ANOVA, followed by least-significant difference post hoc with SPSS 13.0 (SPSS, Chicago, IL, USA). A p value of <0.05 was considered to be statistically significant.

## Results

### Assessment of T2DM rat model

Healthy male Sprague–Dawley (SD) rats were fed with a high-fat diet. After 4 weeks, the high-fat diet resulted in a significant increase in body weight and hyperinsulinemic (p < 0.05), which suggests that rats have already developed obesity and insulin resistance. Injection of STZ significantly increased RBS levels in T2DM rats (p < 0.05), thus producing hyperglycemia (Table 2). FSI levels and the body weight were reduced slightly after STZ injection but remained higher than that of controls (p < 0.05) (Table 2). Meanwhile, the rats in T2DM group appeared polydipsia and polyphagia.

**Table 2:** **Effect of high**-**fat diet and STZ injection on general characteristics of type 2 diabetic rats**

**Parameters**	**Cont group**		**T2DM group**	
	**Before injection**	**After injection**	**Before injection**	**After injection**
Body weight (g)	344.09 ± 16.56	350.20 ± 21.07	383.10 ± 17.80 ^*^	377.54 ± 17.66 ^*^
Food intake (g)	29.02 ± 2.65	29.90 ± 2.16	29.13 ± 3.25	34.39 ± 1.85 ^*^
Water intake (ml)	48.60 ± 3.50	43.90 ± 1.35	46.10 ± 5.27	61.72 ± 4.82 ^*^
Rectal temperature (°C)	37.01 ± 0.41	37.11 ± 0.38	37.15 ± 0.59	37.48 ± 0.33
RBS (mmol/L)	4.96 ± 0.27	5.50 ± 1.04	5.13 ± 0.26	21.35 ± 4.20 ^**^
FSI (mIU/L)	13.70 ± 2.10	13.17 ± 4.65	33.27 ± 7.18 ^*^	20.55 ± 8.15 ^*^

Data are given as mean ± SD., ^*^p < 0.05, ^**^p < 0.01 vs. the Cont group. *RBS* random blood sugar, *FSI* fasting serum insulin.

**Figure 1:**
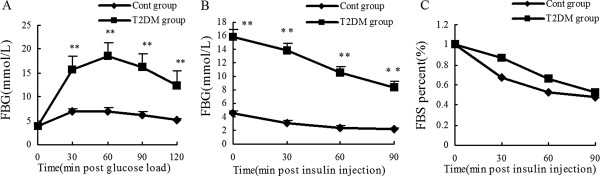
**Assessment of the type 2 diabetic rat model. (A)** Fasting blood-glucose levels following glucose challenge (50%, 2 g/kg) performed after STZ injections. **(B)** Fasting blood-glucose levels following insulin challenge (0.75 IU/kg) performed after STZ injections. **(C)** Fasting blood-glucose, represented as the percentage change from baseline, is reduced following insulin challenge (0.75 IU/kg) performed after STZ injection. Data are represented as means ± SD, ^*^p < 0.05, ^**^p < 0.01 vs. the Cont group. FBG, fasting blood-glucose.

T2DM rats exhibited severe hyperglycemia upon administration of glucose and exhibited impaired glucose tolerance following OGTT (Figure 1A). Insulin sensitivity detected by ITT was also impaired in the T2DM rats. (Figure 1B and 1C).

### Improvement of behavioral by JZD

**Figure 2:**
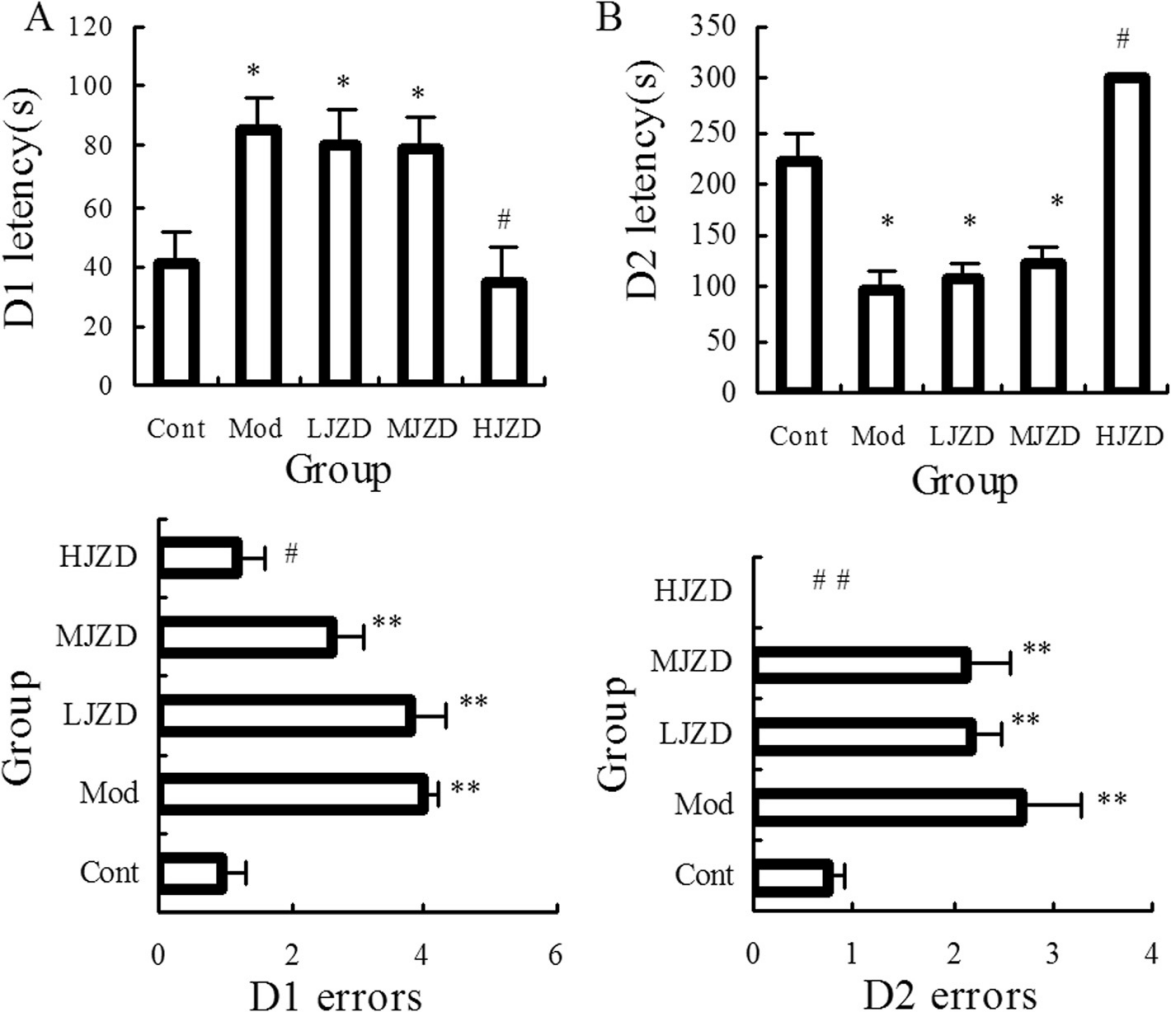
**Effect of JZD on step**-**down test of DACD rats. (A)** Latencies and numbers of errors on Day 1 of the step-down test. Latencies of the Mod group were longer than those of the Cont and HJZD groups. Numbers of errors during the memory acquisition period on Day 1 of the step-down test. The Mod group had more errors than Cont and HJZD groups. **(B)** Latencies and numbers of errors on Day 2 of the step-down test. Latencies of the Mod group were shorter than those of the Cont and HJZD groups. Numbers of errors during the memory consolidation period on Day 2 of the step-down test. The Mod group had more errors than Cont and HJZD groups. None of the HJZD group rats stepped down from the platform on the second day. Data are represented as means ± SD, ^*^p < 0.05, ^**^p < 0.01 vs. the Cont group, ^#^*p* < 0.05, ^# #^*p* < 0.01 vs. the Mod group.

After JZD treatment, behavioral tests were performed. Day 1 of the step-down test was the memory acquisition period. The latency to step-down and numbers of errors were significantly increased in the Mod group compared to the Cont group (p < 0.05) (Figure 2A). JZD treatment improved memory acquisition; there was a significant improvement in both latency time and number of errors in the HJZD group compared to the Mod group (p < 0.05). There was no significant difference in performance between the Cont group and the HJZD group (p > 0.05). Even though in the LJZD and MJZD groups, latency was slightly decreased compared to the Mod group, but the difference was not statistically significant. Memory consolidation was evaluated on D2. The latency to step-down was significantly longer and the number of errors significantly fewer in both the Cont group and the HJZD group compared with the Mod group (p < 0.05). Interestingly, all rats in the HJZD group exhibited latencies of 300 s and all had zero errors during the memory consolidation test (Figure 2B).

**Figure 3:**
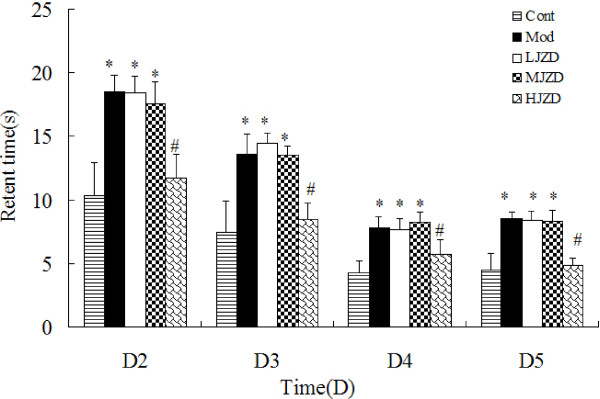
**Effect of JZD on escape latency during the Morris water maze test in DACD rats.** Graphs show mean time ± SD taken to reach the platform (escape latency) for Days 2 through 5. A significant decrease in the escape latency to reach the platform was found on Days 2 through 4 and remained stable on Day 5. Factorial ANOVA showed that the Cont and HJZD groups had a significantly lower retent time on days 2–5 than the other groups. Data are represented as means ± SD, ^*^p < 0.05 vs. the Cont group, ^#^*p* < 0.05 vs. the Mod group.

In the Morris water maze, Day 1 was a habituation session and escape latency from D2 to D5 were statistically analyzed. Figure 3 shows that escape latency to locate the hidden platform was significantly shorter in the Cont and HJZD groups compared to the Mod, LJZD and MJZD groups (p < 0.05). Performance of the Cont and HJZD groups was not statistically different (p > 0.05). Escape latency from D2 through D5 were similar for the Mod, LJZD and MJZD groups and were significantly higher than those for the Cont and HJZD groups (Figure 3). We obtained the similar results in the retention test ,which could suggest spatial memory capacity of rats (Table 3).

**Table 3:** Effect of JZD on Morris water test objective quadrant retention time and frequencies of DACD rats

**Group**	**Cont group**	**Mod group**	**LJZD group**	**MJZD group**	**HJZD group**
Time(s)	12.75 ± 2.05	5.01 ± 1.66 ^**^	7.34 ± 1.02 ^*^	10.92 ± 1.81^#^	10.43 ± 1.46 ^##^
Frequencies	10.00 ± 1.24	5.42 ± 0.90 ^**^	6.18 ± 1.13 ^*^	7.57 ± 1.74 ^#^	8.43 ± 1. 06 ^##^

Data are represented as means ± SD, ^*^p < 0.05, ^**^p < 0.01 vs. the Cont group, ^#^*p* < 0.05, ^##^*p* < 0.01 vs. Mod group.

### Effect of JZD on indicators of hippocampal ERS

**Figure 4:**
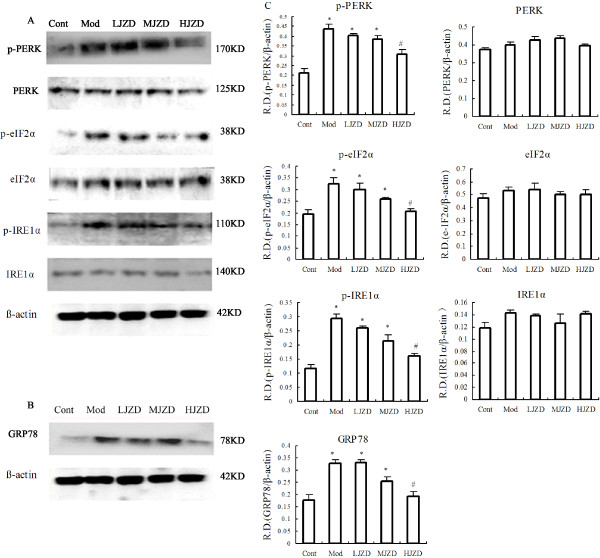
**Effect of JZD on indicators of DACD rat hippocampal ERS. (A)** Representative protein levels of PERK , IRE-1α and eIF2α and their phosphorylated forms from hippocampal tissues of the Cont, Mod and JZD-treated rats were assessed by Western blotting using specific antibodies. **(B)** GRP78 in hippocampal tissues of the Cont, Mod and JZD-treated rats were assessed by Western blotting. **(C)** The relative densities (R.D.) of each protein are represented. The relative densities are obtained by dividing the density of each antibody value by the internal reference β-Actin. Data are represented as means ± SD, n = 5 per group. ^*^p < 0.05, ^**^p < 0.01 vs. the Cont group, ^#^*p* < 0.05, ^# #^*p* < 0.01 vs. the Mod group.

Protein expression levels were determined for several ERS markers, such as PERK, IRE-1α and eIF2α, in hippocampal tissues from rats. The levels of phosphorylated ERS proteins were significantly higher in Mod rats than in the Cont and JZD-treated groups. During the JZD treatment, the expression of phosphorylated ERS indicators was decreased compared to the Mod group, especially in the HJZD group the expression of phosphorylated ERS indicators was decreased significantly (p < 0.05) (Figure 4A).

Under normal conditions, the ER chaperone protein GRP78 binds the transmembrane proteins PERK, IRE-1A, and ATF6 to form an inactive complex [22]. Under conditions of stress, these ERS indicators dissociate from GRP78 and are autophosphorylated and subsequently activated [23]. We detected higher GRP78 levels in hippocampal tissues of the Mod group than those of the Cont group. Treatment with JZD the GRP78 expression level was decreased especially in the HJZD group (p < 0.05) (Figure 4B).

### Effect of JZD treatment on markers of insulin resistance and insulin signaling transduction

**Figure 5:**
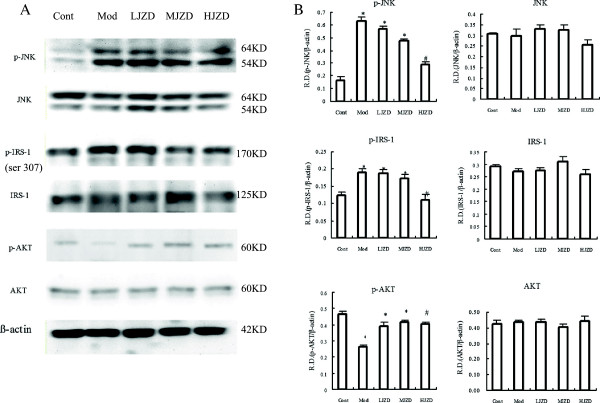
**Effect of JZD on markers of insulin resistance and insulin signal transduction in the hippocampus of diabetic rats. (A)** Representative protein levels of JNK, IRS-1, AKT and their phosphorylated forms from hippocampal tissues of the Cont, Mod and JZD-treated rats were assessed by Western blotting using specific antibodies. **(B)** The relative densities (R.D.) of each protein are represented. The relative densities are obtained by dividing the density of each antibody value by the internal reference β-Actin. Data are represented as means ± SD, *n* = 5 per group. ^*^p < 0.05 vs. the Cont group, ^#^*p* < 0.05 vs. the Mod group. group.

JNK is an important marker of ERS downstream signaling pathways. Insulin resistance is associated with the activation of JNK and subsequent JNK-mediated serine phosphorylation of IRS-1 at Ser-307 that negatively regulates the insulin signaling pathway[24]. AKT is a marker of insulin signaling transduction sensitivity. We found that the levels of p-JNK and p-IRS-1 (ser307) were elevated and p-AKT was decreased in the hippocampus of the Mod group rats compared to the Cont group rats. Treatment with JZD suppressed phosphorylation of JNK and IRS-1(ser307) but increased AKT phosphorylation (Figure 5).

## Discussion

There is a close relationship between T2DM and Alzheimer’s disease (AD). This relationship is perhaps due in part to the effects of sustained hyperglycemia on the central nervous system (CNS), though hypercholesterolemia and hypertension caused by metabolic syndrome can also affect the CNS. These effects result in mild to moderate cognitive dysfunction, decline in learning and memory, or even AD [25]. Recent studies have shown the cognitive decline in the hippocampus of patients with T2DM [26,27]. Furthermore, AD-like pathological changes such as abnormal Aβ deposition and tau hyperphosphorylation were discovered in patients with T2DM [28] and diabetic animal models [29].

Tea as a traditional medicine was used in the Chinese medicine prescription has undergone a very long history period. In ancient China, some famous tea therapy recipes were widely used, which could improve the intelligence and cognitive function. Modern pharmacology research also shows that the main ingredients, polyphenols and theanine, have the function both slowing the aging and improving cognition and neuroprotective functions [30,31]. So polyphenols and theanine were used as the principal drug in the prescription of JZD. Ginsenosides have the effects of benefiting qi, restoring pulse and treating collapse, tonifying the spleen and lung, promoting the production of body fluid to quench thirst, tranquilizing the mind and improving cognition. Ginsenosides have also been demonstrated to have neuroprotective effects in vitro [32] and in vivo [33] and have been shown to improve behavioral deficits. Polygala has the effects of relieving mental distress, tranquilizing the mind, removing phlegm to induce resuscitation, treating carbuncles and dissolving lumps. Lastly in animal experiments[14,34], polygalic acid could significantly ameliorate memory impairment, particularly in short-term memory, which is most affected in the early stages of AD. So ginsenosides and polygalic acid were used as the assistant drug in the prescription of JZD. In accordance with theories of TCMs, JZD has the therapeutic effects of including replenishing qi, tranquilizing the mind and improving the cognitive function. Moreover, JZD has already been utilized in the clinical setting to treat patients with AD or mild cognitive impairments with good results. In scopolamine-induced AD mouse models, we had previously demonstrated improvements in cognitive function with JZD (data not shown). Given the close associations between AD and DACD, we hoped to verify the therapeutic effects of JZD on DACD in T2DM rats.

We induced T2DM in rats with a high-fat diet and low-dose STZ injections [16]. First, rats fed a high-fat diet develop insulin resistance and obesity, then low-dose STZ induced a mild impairment of insulin secretion that was similar to a feature of T2DM. This model would closely mimic the natural history and metabolic characteristics of T2DM in human [34]. Cognitive deficits gradually begin within eight weeks of diabetes induction and reached a maximum after 12 weeks [35]. We tested cognitive performance after setting up T2DM model 12 weeks with the step-down test and the Morris water maze (MWM). These two tests are common utilized for evaluating learning and memory in rats and mice [36]. The step-down test is a classic model behavioral test. In our study, we observed a significant increase in step-down latency and numbers of errors on D1 of the step-down test in DACD rats, suggesting memory acquisition capacity impairment in these animals. The step-down latency was significantly shorter and the numbers of errors were significantly more on D2 of the step-down test in DACD rats, suggesting memory consolidation capacity impairment in these animals. These results are in agreement with other studies that have also verified cognitive impairment in streptozotocin-induced diabetes mellitus [37,38]. However, when the DACD rats were treated with JZD, the step-down latency in the step-down test was similar to that found for rats from the control group. These findings indicate that treatment with JZD was able to prevent learning and memory impairment induced by diabetes. The MWM is a test of spatial learning and reference memory. The DACD rats showed enhanced spatial learning and memory in the MWM behavioural testing. The JZD-treated rats had a lower latency to reach the hidden platform from day 2 onwards; the decrease reached a significant level on day 5. This was in accordance with previous studies [18,35,39,40].

One important link between AD and T2DM is insulin resistance in the CNS due to alterations in insulin receptor sensitivity and insulin signaling transduction. These alterations affect the expression and metabolism of Aβ [41] and tau proteins [42] and also affect synaptic plasticity [43] and neural cell degeneration processes [44] involved in cognitive function [45]. For this reason, we used the T2DM rat model to investigate the mechanisms of DACD as well as CNS insulin resistance and insulin signaling transduction. In our research, impairment of insulin signaling transduction in the hippocampus was observed in DACD rats, and the impairment could be improved by JZD. We analyzed the impairment of insulin signaling in the hippocampus in T2DM rats in several different ways. The inhibition of brain insulin phosphatidylinositol 3 kinase/protein kinase B (PI3K/AKT) signaling pathways accelerated Aβ fibrillogenesis by inducing GM1 ganglioside clustering in the presynaptic membranes of primary neurons [46]. Moreover, the insulin-activated AKT phosphorylation antagonized Aβ-induced neural cell apoptosis by restoring the mitochondrial membrane potential [47]. The insulin-resistant state induced by STZ intracerebroventricularly (icv) injection in Tg2576 mice exacerbated AD-like changes, such as spatial cognitive impairment, and increased Aβ and total tau protein levels [48]. Meanwhile, the phosphorylation of the insulin receptor and AKT reduction could increase glycogen synthase kinase-3β (GSK3β) activity. This insulin signaling impairment was associated with a concomitant increase in tau phosphorylation levels [49]. The insulin signal impairment also affects synaptic plasticity and neural cell degeneration involved in cognitive function. Soeda [50] found that inhibition of inositol polyphosphate-5 phosphatase-like 2(SHIP2), a potent negative regulator of insulin/ insulin-like growth factor-I(IGF1) actions in the brain, ameliorated the impairment of hippocampal synaptic plasticity and memory formation in db/db mice. Li [51] reported activating AKT/ERK could induce the neuronal apoptosis in diabetic mice.

As insulin signaling transduction affected cognitive function, we therefore investigated the mechanisms of DACD as well as CNS insulin resistance and insulin signaling transduction in T2DM rats. We found increased levels of the phosphorylated form of the insulin resistance marker IRS-1 ser 307 and decreased levels of the downstream target AKT in T2DM rat hippocampus. Treatment with JZD decreased the IRS-1 phosphorylation levels at ser307 and result in the AKT phosphorylation levels increasing. These results demonstrate that JZD may improve cognitive impairment by reducing the brain’s insulin resistance and promoting recovery of insulin signal transduction.

Under diabetic conditions, the ERS response is induced in various tissues [52], leading to activation of the JNK pathway. In tissues such as liver, adipose tissue, muscle or cardiac myocytes that have become insulin-resistant, ERS promotes JNK-dependent serine phosphorylation of IRS-1, which in turn inhibits insulin signal transduction. Su et al. [53] suggested that hepatic ERS induced by apolipoprotein B100 led to abnormal activation of glycogen synthase, by way of activation of JNK and suppression of the insulin signaling cascade. In cardiomyocytes from ethanol-fed mice. Li and Ren [54] found that serine phosphorylation of IRS-1 decreased phosphorylation of AKT. GSK-3β expression was reduced, JNK was activated and the ERS markers eIF2α, IRE-1α, GRP78 and Gadd153 were upregulated. They concluded that elevated cardiac acetaldehyde exposure may exacerbate alcohol-induced myocardial dysfunction, hypertrophy, insulin insensitivity and ERS. Sreejayan [55] also showed that markers of insulin resistance (phospho-c-Jun and IRS-1 phosphoserine) and ERS (p-PERK, p-IRE-1A, p-eIF2α) were elevated in liver and cultured muscle cells in ob/ob mice. These studies demonstrate that ERS is reduced by various factors and could inhibit the insulin signaling pathway and lead to insulin resistance by activation of phosphorylation of JNK in peripheral organs. Moreover, there is an insulin signaling cascade in the rat hippocampus similar to that described in peripheral tissues[56]. Insulin resistance is present in the brain and decrease glucose metabolism [56]. As a result, neuronal activity in the hippocampus rapidly decreases with hippocampal-dependent learning and memory [57]. So we hypothesized that, under diabetic conditions, ERS is reduced, the insulin signaling pathway is inhibited, and insulin resistance is worsened by activation of JNK in the CNS. We assessed the expression of insulin signal transduction and ERS by Western blotting in the hippocampus in the T2DM rat model to verify our hypothesis. In our study, T2DM rats showed activation of ERS, as demonstrated by the increased expression of phosphorylated ERS hallmarks. They also demonstrated impairment of insulin signal transduction, resulting in increased activation of JNK, increased serine phosphorylation of IRS-1, and decreased function of AKT. Treatment with JZD significantly improved cognitive function of T2DM rats. On a molecular level, JZD treatment reduced ERS and improved insulin signal transduction in the hippocampus. These experimental results confirmed our hypothesis. One of the important principles underlying TCMs is that several extracts in combination may play coordinate roles to enhance the treatment effects in complex diseases. However, at present, we do not know which extract is most efficient. We plan to carry out further studies to identify the most effective composition of JZD.

## Conclusions

The results of this study demonstrate that JZD treatment with improved cognitive function in the T2DM-DACD rat model. The possible mechanism for DACD was related with ERS inducing the insulin signal transduction dysfunction in T2DM rats’ hippocampus. The JZD could reduce ERS and improve insulin signal transduction and insulin resistance in T2DM rats’ hippocampus and as a result improved the cognitive function.

## Abbreviations

Aβ: Amyloid protein; AD: Alzheimer’s disease; AKT: Protein kinase B; CNS: Central nervous system; DACD: Diabetes-associated cognitive decline; DE: Diabetic encephalopathy; eIF2α: α subunit of translation initiation factor 2; ERS: Endoplasmic reticulum stress; FSI: Fasting serum insulin; GRP78/Bip: Molecular chaperone 78-kD glucose-regulated protein; GSK3β: Glycogen synthase kinase-3β; IGF1: Insulin-like growth factor-I; IRS-1: Insulin receptor substrate-1; IRE1: Inositol-requiring enzyme-1; ITT: Insulin tolerance tests; JNK: Jun NH2-terminal kinases; JZD: Multi component chinese medicine Jinzhida; MWM: Morris water maze; OGTT: Glucose tolerance tests; PERK: Phosphorylated protein kinase RNA-like endoplasmic reticulum kinase; PI3K/AKT: Phosphatidylinositol 3 kinase/protein kinase B; RBS: Random blood sugar; SD: Sprague–dawley; SHIP2: Inositol polyphosphate-5 phosphatase-like 2; TCMs: Traditional chinese medicines; T2DM: Type 2 diabetes mellitus.

## Competing interests

The author(s) declare that they have no competing interests.

## Authors’ contributions

Conceived and designed the experiments: LBZ. Performed the experiments: XHC, LNL, XS, ZLF, MJW, SH, LPZ and JS. Analyzed the data: XQ, FLZ, and XGL. Contributed reagents/materials/analysis tools: CCB, NL, and GZH. Wrote the paper: XHC, XHC and LNL are equivalent contributors. All authors read and approved the final manuscript.

## Pre-publication history

The pre-publication history for this paper can be accessed here:

http://www.biomedcentral.com/1472-6882/13/161/prepub
